# Associations of the Food Insecurity Experience Scale with Socioeconomic and Psychological Factors in Japan

**DOI:** 10.3390/nu17223536

**Published:** 2025-11-12

**Authors:** Rei Fujiwara, Ryoko Katagiri, Takahiro Tabuchi

**Affiliations:** 1 Research Institute of Life Innovation, Toyo University, 1-7-11 Akabanedai, Kita-ku, Tokyo 115-8650, Japan; rei510w40@gmail.com; 2 Faculty of Informatics, Graduate School of Informatics, Chiba University, 1-33 Yayoi-cho, Inage-ku, Chiba-shi 263-8522, Chiba, Japan; 3 Division of Epidemiology, School of Public Health, Tohoku University Graduate School of Medicine, 2-1, Seiryoucho, Aoba-ku, Sendai 980-8575, Miyagi, Japan; tabuchitak@gmail.com

**Keywords:** food security, food insecurity, Food insufficiency experience scale, food assistance, COVID-19, Japanese

## Abstract

**Background:** The Food Insecurity Experience Scale (FIES), developed by the Food and Agriculture Organization of the United Nations (FAO), is a standardized tool for measuring food insecurity (FI) and enabling international comparisons. Although reliable tools are essential, the FIES has not yet been applied or validated in research conducted in Japan. This study aimed to assess the internal validity of the FIES among Japanese individuals and to identify the sociodemographic, socioeconomic, and psychological characteristics and public assistance status of individuals experiencing FI. **Methods:** A large-scale, cross-sectional online survey targeting Japanese adult panel members was conducted in 2022. In total, 23,576 respondents were included in the final analysis. The internal validity of the FIES was evaluated according to FAO guidelines. Multivariate-adjusted odds ratio (AOR) and 95% confidence interval (CI) were calculated to determine the associations between FI and sociodemographic factors as well as other factors. **Results:** The FI scale in this population was acceptable, as indicated by infit statistics ranging from 0.7 to 1.3. Reliability was adequate (0.72). Additionally, the number of types of public assistance (AOR [95% CI] = 1.17 [1.00–1.47]) and factors such as greater severity of psychological distress (AOR [95% CI] = 5.89 [4.74–7.33]) were significantly associated with a higher risk of FI. **Conclusions:** This study confirmed the reliability and internal validity of the FIES in a Japanese population and identified characteristics of groups at high risk of FI. Focusing on these populations may help detect previously overlooked FI in developed countries and enable timely interventions.

## 1. Introduction

According to the World Food Summit in 1996, food security means that all people always have physical and economic access to sufficient, safe and nutritious food that meets their dietary needs and food preferences for an active and healthy life [1]. Ensuring a stable food supply for citizens and achieving “food security” have become critical policy challenges worldwide. However, the global prevalence of moderate or severe food insecurity (FI) dramatically increased in 2020, and approximately 2.3 billion people, or nearly 30% of the world’s population, experienced moderate or severe FI in 2023 [2]. Coronavirus disease 2019 (COVID-19) is a major factor behind the dramatic increase in FI in 2020 [3]. Even in high-income countries, the impact on vulnerable groups, such as low-income groups, has been severe [4,5,6,7,8,9].

Monitoring and comparing the FI across regions and countries is difficult because it arises from diverse and complex situations. The Food and Agriculture Organization of the United Nations (FAO)–Voices of the Hungry project has developed the Food Insecurity Experience Scale (FIES) as a tool to assess food access; it represents one of the four FAO—defined food security dimensions (availability, access, utilization, and stability) [10,11]. The FIES measures of FI at both individual and household levels [12]. Furthermore, Goal 2.1 of the Sustainable Development Goals calls for the elimination of moderate or severe food supply shortages using the FIES as an indicator. Against this background, the FIES has been widely utilized as a globally standardized tool for assessing FI [10].

Studies examining the validity of the FIES have been conducted in various countries, mainly in low- and middle-income settings, both through FAO monitoring and independent research exploring its validity and related factors [13,14,15,16,17,18,19]. These studies have demonstrated that FI, as assessed by the FIES, is associated with various socioeconomic factors, including low income [13,14,15,16], low education [13,14,15], unemployment [13,15], and the number or ages of cohabiting children [13,15]. In addition, associations with health outcomes have been reported, such as non-communicable diseases [14,18] and mental health [17,19]. In contrast, large-scale studies conducted in high-income countries remain limited. A study conducted in the United Kingdom examined the prevalence and associated factors of food insecurity using the FIES in a nationally representative sample (*n* = 2000) [20]. The results showed that approximately 3% of respondents experienced severe food insecurity, with higher prevalence observed among younger individuals, those with lower income, and tenants in rented housing. By contrast, no significant regional differences were reported.

According to World Bank data from 2022, the prevalence of moderate or severe FI in Japan was 5.5% [21]. Furthermore, data from the Organisation for Economic Co-operation and Development (OECD) indicate that Japan’s poverty rate was approximately 16% in 2021, exceeding the levels observed in many other OECD member countries [22]. These findings suggest that, despite Japan’s status as a high-income country, socioeconomic disparities remain considerable. In addition to these widening disparities, the COVID-19 pandemic may have further exacerbated FI among vulnerable populations by causing employment instability and income loss. Consequently, FI may increase the risk of nutritional deficiencies and mental health problems, particularly among low-income and socially disadvantaged groups. Moreover, according to the Ministry of Agriculture, Forestry and Fisheries, Japan’s food self-sufficiency rate (calorie-based) is among the lowest of all developed countries (38% in 2022), reflecting the country’s heavy dependence on food imports [23]. Therefore, climate change, including extreme weather events and global production instability, may pose a significant threat to Japan’s food security.

Validity for use in research populations in Japan has not yet been examined. Therefore, comparing and evaluating the sociodemographic characteristics of individuals experiencing FI in Japan using international standards is difficult. Although FI is considered a major issue in low-income countries, it may affect low-income populations in high-income countries, where its relatively low prevalence often leads to it being overlooked. Identifying the factors associated with FI in countries, such as Japan, can provide empirical evidence to support food access during emergencies, such as the COVID-19 pandemic. This evidence can be useful for policy development and intervention planning in high-income countries.

This study aimed to assess the internal validity of the FIES in Japan and to identify the sociodemographic, socioeconomic, public assistance status, and psychological distress factors associated with vulnerable populations experiencing FI.

## 2. Materials and Methods

### 2.1. Study Design and Participants

We used data from the Japan COVID-19 and Society Internet Survey (JACSIS) study. The JACSIS study is a series of cross-sectional surveys assessing the conditions of life, health status, and social and economic activities of the population, including those related to the COVID-19 pandemic. Details of the JACSIS are provided in another study [24]. The JACSIS is a nationwide online cohort study conducted in Japan from August 2020 to the present. The survey used a random sampling strategy, stratified by sex, age, and region to cover a broad range of demographics, ensuring representation across all 47 prefectures of Japan. The survey was conducted using a web-based self-report questionnaire administered by a major Internet research firm (Rakuten Insight, Inc., Tokyo, Japan). This research firm is one of the largest in Japan, with approximately 2.2 million registered panelists as of September 2022. We based our analysis on the survey data collected in 2022. Participants were recruited from among panel members aged 16–79 years of an Internet research firm between 29 September and 19 October 2022. In total, 32,000 individuals participated in the survey. Among these, we excluded respondents who appeared to have provided irregular or inconsistent responses (*n* = 3370) according to an algorithm developed by the JACSIS study group to maintain data quality [25].

**Figure 1:**
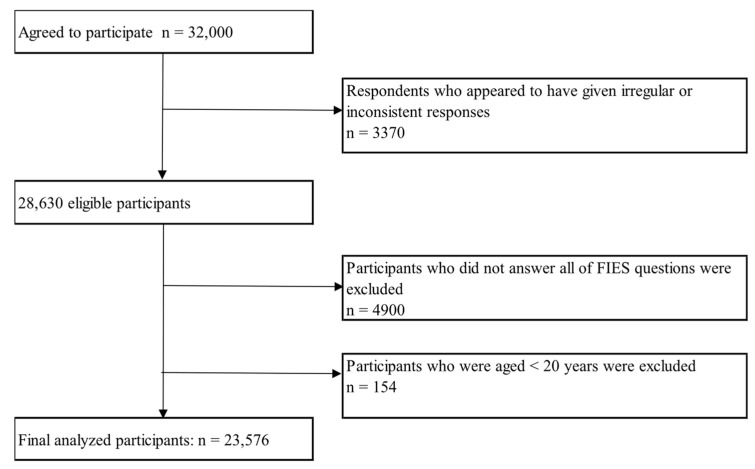
Flowchart of participant selection.

Participants who did not answer all FIES questions (*n* = 4900) or who were aged < 20 years (*n* = 154) were excluded, as this analysis focused on adults. In total, 23,576 participants were included in the analysis. The participant selection process is illustrated in Figure 1.

### 2.2. Food Insecurity Experience Scale (FIES)

The FIES consists of eight questions related to food access. The respondents answered eight questions using four response options (1. Yes, 2. No, 3. Don’t know, and 4. Refused). The participants were instructed to reflect on their personal experiences over the past 12 months. The official Japanese version of the FIES, developed by FAO, was originally designed for interviewer administration. Therefore, in this study, we made minor modifications to adapt it for use in an online Japanese-language survey. Crimson Interactive Pvt. Ltd. (Mumbai, Maharashtra, Ulatus, www.ulatus.jp) was commissioned to translate the FIES into Japanese, and the FIES was translated using a back-translation step. The eight questions and their corresponding labels are presented in Table 1.

### 2.3. Statistical Validation of FIES Data

All statistical validations of the FIES data were performed in accordance with the recommendations of the FAO e-Learning Academy course guidelines [26]. Responses to each item were coded for aggregation of the FIES data. A score of 1 was given for “1. Yes” and a score of 0 for “2. No.” Additionally, “3. Don’t know” and “4. Refused” responses were treated as missing, and cases with missing responses for any FIES item were excluded entirely from the analysis [26]. The FIES scores ranged from 0 to 8, with higher values indicating more severe FI.

According to the FAO method [26], we used Rasch analysis based on the principles of item response theory to analyze item fit statistics (Infit and Outfit statistics), the residual correlation matrix, and Rasch reliability. Rasch analysis is used to analyze the responses to surveys or test items [27]. Rasch analysis was performed using the R package (RM.weights), which is freely available from the FAO [28]. This method is suitable for evaluating the validity of the FIES [10,29,30,31]. Infit statistics are instrumental in identifying items that do not function adequately within a specific population, whereas outfit statistics are particularly useful for detecting outliers. Acceptable thresholds for item fit statistics are typically defined as infit values ranging from 0.7 to 1.3 and outfit values < 2 [26,30]. A residual correlation evaluates the independence of responses for each item; if the residual correlation between two items ≥ |0.4|, responses to that item are considered redundant and rated as having a weak ability to accurately measure FI [30]. Rasch reliability assesses the discriminatory power of the overall scale by measuring the proportion of variability in the data explained by the Rasch model. Moreover, a Rasch reliability value > 0.7 is acceptable [10].

The FAO developed two FIES-based indicators for global monitoring: the proportion of the population experiencing moderate-to-severe FI (FImod + sev) and the proportion of the population experiencing severe FI (FIsev). We used the FIES Excel template recommended by the FAO to calculate these two indices and performed the equating process [28]. The equating process was performed to calibrate the measurements obtained from specific application of the experience-based food security questionnaires to the global FIES reference scale and to calculate the prevalence of FImod + sev and FIsev.

### 2.4. Sociodemographic and Socioeconomic Variables, Including Public Assistance

Data on sex, age, educational level, employment status, marital status, number of people in the household, household income in 2022, and public assistance status were collected using a questionnaire. The following variables were created: sex; age group (20–30, 31–40, 41–50, 51–60, 61–70, and ≥71 years); educational level (high school or below, 2-year college graduate or technical school, and university and above); employment status (full-time employee/self-employed, part-time, retired/homemaker/student and unemployed); marital status (married, unmarried, and divorced/deceased); number of people in the household (including themselves); annual household income (<1,000,000 yen, 1,000,000–<5,000,000, 5,000,000–<10,000,000, and ≥10,000,000); number of types of public assistance (number receiving public assistance from April 2022 to present 0, ≥1); and types of public assistance (the respondents were instructed to select all public assistance programs involving financial support received from April 2022 to present). Table 2 shows the 10 public assistance schemes and provides brief descriptions of each.

### 2.5. Severity of Psychological Distress

To confirm the relationship between the FIES and the severity of psychological distress, the Japanese translation of the Kessler Psychological Distress Scale (K6) was used [32,33]. The K6 consists of six questions assessing the severity of psychological distress, and the response options are scored from 0 to 4 on a 5-point scale (total score range: 0–24). In this study, based on the Comprehensive Survey of Living Conditions conducted by the Ministry of Health, Labour and Welfare of Japan, K6 total scores were divided into groups of 0–4 (normal), 5–9 (possible mild mood or anxiety disorder), and >10 (mood or anxiety disorder), and these were used for analysis [34].

### 2.6. Statistical Analyses

Data were analyzed using the statistical software package STATA version 18.0 (StataCorp, College Station, TX, USA). Given the potential discrepancy between Internet survey respondents and the representative Japanese population, the analysis used the inverse probability weighting method with propensity scores (PS) to adjust for any bias introduced by the Internet survey [35]. The PS was calculated using a logistic regression model with sex, age, and socioeconomic factors from the pooled dataset of the Comprehensive Survey of Living Conditions of People on Health and Welfare, which is considered a nationally representative sample of the Japanese population. In this study, FI status was defined as “Food security” if the FIES score was 0 and “FI” if the FIES score was 1–8. In addition, additional analyses were conducted using the severity-based categories of the FIES recommended by the FAO: Food security/mild: scores 0–3, moderate: scores 4–6, severe: scores 7–8 [26]. As this study aimed to examine factors associated with FI within Japan rather than conduct international comparisons, crude FIES scores were used instead of the globally equated values recommended by the FAO. Weighted percentages of variables were expressed according to FI status. To examine the multivariate-adjusted odds ratio (AOR) and 95% confidence interval (CI) for each factor comparing “Food Secure” compared with “Food Insecure,” participants, a weighted logistic regression model was constructed. Sociodemographic, socioeconomic, number of types of public assistance, and severity of psychological distress variables, were used as dependent variables, and FI status was used as the independent variable. Variables other than the dependent variables were considered confounders. *p*-values for each factor were calculated using the χ^2^ test between the “Food Security” and “FI” groups. The potential confounding factors considered were sex, age, educational level, employment status, marital status, number of people in the household, household income, and number of types of public assistance. The severity of psychological distress was not included as a confounding factor because of the potential for over adjustment [36]. A few participants did not answer questions regarding educational level (*n* = 96) or annual income (*n* = 4115). Missing values were categorized as a separate group and included in the regression models to estimate odds ratios. Additionally, stratified analyses by sex were performed to investigate sex-specific associations. *p*-value < 0.05 was considered statistically significant. Participants were excluded when the percentages of their educational level and annual income were calculated.

### 2.7. Ethics

This study adhered to the principles outlined in the Declaration of Helsinki, and all study procedures were approved by the Ethics Committee of the National Cancer Center, Japan (approval no. 2020-447, approved on 22 January 2021) and the Osaka International Cancer Institute (approval no. 20084, approved on 19 June 2020). Written informed consent was obtained from all participants, including those aged < 20 years. Additionally, as this study was noninvasive, approval was granted by the institutional review boards, ensuring that the study purpose was made public and that participants had the opportunity to opt out if they wished.

## 3. Results

### 3.1. Item Parameters and FIES of Item Fit Statistics

Table 3 shows the item parameters and the FIES item fit statistics. Infit statistics ranged between 0.7 and 1.3 for all items, meaning they were within the acceptable range for the Rasch model. Outfit statistics were slightly higher than the maximum allowed value (<2) for the “WORRIED” item. The values for the residual correlations, average Rasch reliability, FImod + sev, and FIsev were as follows (these values are not presented in Table). The residual correlation was 0.015–0.361, the mean Rasch reliability was 0.72, and the estimated prevalences were 3.41% for FImod + sev and 1.15% for FIsev.

### 3.2. Association Between Socioeconomic and Psychological Characteristics and Food Insecurity Experience Scale Score

Table 4 shows the participants’ characteristics based on their FIES scores. Factors associated with the FIES scores included age, educational level, marital status, annual household income, public assistance status, and severity of psychological distress. Among all participants, compared with the 41–50 age group (reference), the 20–30 and 31–40 age groups had significantly higher odds of experiencing FI (AOR [95% CI] = 2.11 [1.56–2.85] and 1.52 [1.17–1.97], respectively). In contrast, participants in the 61–70 and ≥71 age groups had a significantly lower risk (AOR [95% CI] = 0.26 [0.16–0.44] and 0.26 [0.15–0.44], respectively). Compared with the university degree or higher group (reference), the 2-year college graduate or technical school group had significantly higher odds of experiencing FI (AOR [95% CI] = 1.24 [1.01–1.53], respectively). Compared with the annual income of ≥5,000,000 and <10,000,000 yen group (reference), those with an annual household income of <1,000,000 yen had a significantly higher risk of experiencing FI (AOR [95% CI] = 3.60 [2.33–5.54]), as did those with an annual household income between 1,000,000 and 5,000,000 yen (AOR [95% CI] = 2.11 [1.69–2.64]). Regarding public assistance status, those who received at least one form of public assistance were at higher risk of FI than those who did not (AOR [95% CI] = 1.17 [1.00–1.47]). In addition, participants who reported receiving public assistance in the form of an employment adjustment subsidies, business sustainability subsidies, housing security benefits, public assistance (welfare), child-rearing allowance, and other allowances were significantly more likely to experience FI than those who did not receive such assistance. Severity of psychological distress was strongly associated with FI. Compared with the normal group (reference), the possible mild mood or anxiety disorder and mood or anxiety disorder groups were at significantly higher risk of FI (AOR [95% CI] = 2.80 [2.19–3.59] and AOR = 5.89 [4.74–7.33], respectively). Among all participants, the results were generally consistent with the sex-stratified analyses, showing no major differences between males and females overall. However, employment status differed by sex: compared with males in full-time or self-employed work (reference), those in part-time employment had significantly higher risk of experiencing FI (AOR [95% CI] = 1.47 [1.02–2.12]). No significant differences were observed among females.

### 3.3. Association Between Sociodemographic, Socioeconomic, Public Assistance Status, and Severity of Psychological Distress Characteristics and Food Insecurity Experience Scale Score Recommended by the FAO

In addition, this study analyzed the associations between FI and sociodemographic and other related factors using severity-based categories of the FIES (Food security /mild: scores 0–3, Food Moderate: scores 4–6, Food Severe: scores 7–8), as recommended by the FAO (Table S1). The results showed that the associations between FI and sociodemographic and other related factors were consistent across the different levels of severity.

## 4. Discussion

This is the first study to verify the internal validity of the FIES in Japan and examine the association between the FIES scores and factors such as sociodemographic, socioeconomic, public assistance status, and severity of psychological distress using a large dataset. By applying the Rasch model to assess the quality of the collected data, we confirmed that the FIES fit the model assumptions. In this study, younger age, lower educational level, lower income, receipt of public assistance, and greater severity of psychological distress were associated with the risk of FI. Table 1 shows the item parameters and the FIES item fit statistics. According to the FAO instructions [26,30], infit statistics between 0.7 and 1.3 are considered acceptable, and all items in this study fell within this range. Outfit statistics were slightly higher than the maximum allowed value (<2) for the “WORRIED” item. One reason for the high outfit values may be that some respondents misunderstood the questions. Although problematic items may need to be removed or reworded, if the infit statistics are within a normally sufficient range, a high outfit value will have a low impact on the validity [29]. Therefore, we included “WORRIED” as it is in the questionnaire in this study. The residual correlation was 0.015–0.361 and was sufficiently low (<0.4), indicating that each item captured a unique aspect of FI [30]. The mean Rasch reliability was 0.72, which was within the acceptable range reported internationally and indicated that the scale effectively differentiated between respondents with different degrees of FI [10]. Although caution is warranted in interpreting the estimates due to the nature of the Internet-based survey, the observed proportions were 3.41% for FImod + sev and 1.15% for FIsev. According to the FAO, the prevalence of FI in each country in 2022 was 24.7% for FImod + sev and 9.7% for FIsev in Asia. In North America and Europe, the FImod + sev is 8.5% and the FIsev is 1.5%, while the global averages of FImod + sev and FIsev worldwide are 28.6% and 10.8%, respectively [38]. When this survey was conducted in 2022, despite the ongoing COVID-19 pandemic in Japan, the prevalence of FI remained relatively lower compared with global levels. However, this study suggests a population with severe FI may exist. Furthermore, in Japan, the subsequent increase in prices may have exacerbated FI (the consumer price indices in Japan were 108.5 and 100 in 2024 and 2020, respectively) [39]. Therefore, it would be inappropriate to refrain from implementing measures based solely on the overall low prevalence of FI. Targeted interventions are necessary, particularly in high-risk groups with severe FI. In addition, food security is also defined as encompassing four dimensions: access, availability, utilization, and stability. This study focused on food access, which may be the first aspect to be affected at the individual level in Japan. Nevertheless, future research should adopt a more comprehensive approach by examining all four dimensions to achieve a deeper understanding of FI.

The younger adult groups (20–30 and 31–40 years) showed a significantly higher risk of FI than the middle-aged group (41–50 years). The 20–30 age group had the highest odds ratio. This may be because of the economic instability of young adults. Young adults, even those with education, have limited sources of income and are at an increased risk of FI [6,40,41,42]. For example, a systematic review examining factors related to FI among college students reported a 35–40% FI rate among college students, putting them at a higher risk of FI than the general population [42]. The older groups (61–70 and ≥71 years) had a significantly lower risk of FI than the middle-aged group. This is because older adults generally tend to have a lower appetite than middle-aged adults; therefore, even if a meal is inadequate for middle-aged adults, it may be adequate for older adults, who may report food security. Another possibility, as Gundersen and Ziliak noted, is the difference in mortality rates [43]. Middle-aged adults with FI may have a higher mortality rate than those without FI [44], owing to a higher risk of developing chronic disease. Thus, older adults may include people with a low risk of FI. For age-appropriate public health policies, it is important to strengthen the assessment of FI, especially in younger adults, and carefully assess the appetite and nutritional status of older adults. In this study, the risk of FI was higher for those with lower educational levels and annual household incomes. Similar results were reported in previous studies using the FIES as a measure of FI [4,13,15,20]. Sex differences were observed in relation to employment status: part-time employment among males was associated with an increased risk of FI, whereas no significant association was found among females. This finding may reflect the fact that males are more likely to be positioned as primary earners within households in Japan, and instability in their employment status is therefore more directly linked to household economic conditions and food access. In contrast, females’ employment status tends to vary depending on life stage and family structure, and their income does not always function as the main financial resource of the household, which may explain why differences in employment status alone do not sufficiently account for FI risk among females. These results suggest that FI countermeasures should not treat sex as a uniform factor but rather take into account the relative contribution of individual earnings to household income and the broader household context, including family structure. The results also indicate that those receiving public assistance were at a higher risk of FI than those not receiving public assistance. Furthermore, analysis based on the type of public assistance indicated that those receiving grants targeted at people with extreme financial needs, such as housing security benefits and public assistance (welfare), were at a higher risk of FI. This suggests that even with assistance, recipients remain in a difficult economic situation, which can further exacerbate FI if assistance is inadequate.

Furthermore, in this study, if the severity of psychological distress assessed by the K6 corresponded to mood or anxiety disorder, the likelihood of FI (FIES score 1–8) was 6.1 times higher than that of food security (FIES score 0) compared with the normal group. However, owing to the nature of the study design, it is unclear whether mental health problems negatively affect overall life and cause FI or whether FI worsens mental health. A systematic review examining the causal relationship between FI and mental health reported that approximately 67% (six out of nine studies) of individuals who experienced FI at baseline reported worse mental health outcomes at follow-up. In contrast, in all (100%) of the seven studies on individuals with poor mental health at baseline, the risk of FI increased in the subsequent follow-up study. From this, it can be concluded that FI and mental health are not unilaterally related but rather bidirectionally [45]. Therefore, when screening for the risk of FI using the FIES, it is desirable to screen for mental health at the same time. Furthermore, given the bidirectional relationship between FI and mental health, incorporating mental health support into food assistance policies may be effective. For example, a more comprehensive intervention for people with FI can be achieved by combining the provision of mental health care and psychological support programs with food distribution and financial assistance. Future studies should include longitudinal and interventional studies to elucidate a causal relationship between FI and mental health. Moreover, it is important to conduct screening using the FIES to contribute to the development of more practical interventions.

The strengths of this study are that it validates the FIES using a large dataset covering all prefectures in Japan and, for the first time in Japan, identifies factors associated with FI as assessed by the FIES. Another strength is that the survey was conducted between September and October 2022 during the COVID-19 pandemic. This study serves as a benchmark for the experience of FIs during the COVID-19 pandemic in Japan. However, this study has certain limitations. First, although this study covered all 47 prefectures and applied inverse probability weighting using propensity scores based on population distributions to enhance representativeness, the study population (*n* = 23,576) still accounted for only a small proportion of Japan’s total population, which may limit the generalizability of the findings. Second, we used household income rather than individual income in the analysis. Household income may not accurately reflect an individual’s economic situation, as it does not account for differences in household size, composition, or the distribution of resources among members. Consequently, the analysis may not fully capture the relationship between personal income and individual behaviors or food access. Future research should incorporate both individual and household income to provide a more comprehensive understanding of how economic status is associated with FI. Third, this study used a weighted model to correct for bias in the Internet survey, but those who did not have access to the Internet were not included in the survey, which may have introduced selection bias. Fourth, although the FIES offers substantial advantages as a globally standardized measure of FI and this study used an anonymous Internet-based survey to minimize desirability response bias, its reliance on self-reported data makes it susceptible to recall and social desirability biases. Moreover, certain items, such as “WORRIED,” may be interpreted differently across cultural contexts, which could influence item fit and cross-cultural comparability. Finally, because this study had a cross-sectional design, it was impossible to identify causal relationships, such as FI and sociodemographic factors.

## 5. Conclusions

The reliability and validity of the FIES in Japan were within an acceptable range. The FIES demonstrated sufficient ability to adequately discriminate between respondents with different FI severities. Factors associated with FI risk include young age, low income, and severe psychological distress. This study shows that the FIES can be used as an effective tool to identify populations at high risk of FI in Japan. Furthermore, by identifying the characteristics of groups at high risk of FI, it is possible to implement nationwide screenings using the FIES, focusing on these groups, and develop more effective food assistance intervention strategies.

## Figures and Tables

**Table 1:** FIES questions and ltems for each question.

Questions: Now I Would Like to Ask You Some Questions About Food.	Item
Q1. During the last 12 months, was there a time when you were worried you would not have enough food to eat because of a lack of money or other resources?	WORRIED
Q2. Still thinking about the last 12 months, was there a time when you were unable to eat healthy and nutritious food because of a lack of money or other resources?	HEALTHY
Q3. During the last 12 months, was there a time when you ate only a few kinds of foods because of a lack of money or other resources?	FEWFOOD
Q4. During the last 12 months, was there a time when you had to skip a meal because there was not enough money or other resources to get food?	SKIPPED
Q5. Still thinking about the last 12 months, was there a time when you ate less than you thought you should because of a lack of money or other resources?	ATELESS
Q6. In the past 12 months, was there a time when your household ran out of food because of a lack of money or other resources?	RUNOUT
Q7. In the past 12 months, was there a time when you were hungry but did not eat because of a lack of money or other resources for food?	HUNGRY
Q8. During the last 12 months, was there a time when you went without eating for a whole day because of a lack of money or other resources?	WHLDAY

**Table 2:** Public assistance schemes and brief descriptions of each.

Public Assistance Schemes	Brief Description of the Schemes
1. Employment adjustment subsidy	This program provides subsidies to employers who are forced to scale down their business operations due to the impact of COVID-19 and implement employment adjustment in order to retain their employees.
2. Public benefit for families with children	This measure provides temporary financial support to households with children, with the aim of mitigating the impact on these households and supporting their consumption.
3. Subsidy for sustaining business	This program provides grants to businesses that have been particularly affected by the spread of COVID-19.
4. Housing security Benefit	This program provides rent-equivalent benefits for a fixed period to individuals who are economically distressed and either have lost their housing or are at risk of losing it.
5. Public assistance (welfare)	This program is designed to guarantee the minimum standard of living as stipulated by the Constitution for individuals facing financial hardship, while also actively supporting their efforts to achieve self-reliance.
6. Unemployment benefit	This benefit is provided to help job seekers maintain a stable livelihood while aiming to return to work as quickly as possible.
7.Disability benefit	This support provides financial assistance from the national or local government to individuals who face difficulties in daily life or employment due to a disability.
8. Care allowance	The “care allowance” is a financial benefit or allowance provided to reduce the burden on family members or others who are caring for elderly or disabled individuals at home.
9. Child rearing allowance	The “child rearing allowance” refers to financial benefits or allowances provided to families raising children, with the aim of offering economic support.
10. Other assistance	Utilizing other public assistance programs that do not fall under 1–9.

**Table 3:** Item parameters and FIES of item fit statistics (*n* = 23,576).

Item	Item Severity	SE	Infit	Outfit
WORRIED	−1.723	0.062	1.246	2.163
HEALTHY	−1.023	0.064	0.862	0.876
FEWFOOD	−0.885	0.064	0.795	0.776
SKIPPED	0.771	0.080	0.946	0.991
ATELESS	−0.805	0.065	0.899	0.875
RUNOUT	0.831	0.080	0.927	1.095
HUNGRY	0.513	0.076	1.022	1.017
WHLDAY	2.323	0.108	1.147	1.604

FIES: Food Insecurity Experience Scale. SE: Standard error of item severity.

**Table 4:** Association between socioeconomic and psychological characteristics and Food Insecurity Experience Scale score (*n* = 23,576).

	All				Male			Female		
Values (%)	FI Status (FIES Score)		AOR (95% CI) ^§^	*p*-Value *	FI Status (FIES Score)		AOR (95% CI) ^§^	FI Status (FIES Score)		AOR (95% CI) ^§^
	Food Security [0]	Food Insecurity [1–8]			Food Security [0]	Food Insecurity [1–8]		Food Security [0]	Food Insecurity [1–8]	
N	21,804	1772	-		10,487	809	-	11,317	963	-
Number	92.5	7.5	-		92.8	7.2	-	92.2	7.8	-
Number (weighted)	92.2	7.9	-		93.0	7.0	-	91.5	0.8	-
Age				<0.001						
20–30	13.7	29.4	2.11 (1.56–2.85)		11.8	27.8	2.29 (1.54–3.41)	15.6	27.1	1.94 (1.26–2.98)
31–40	20.5	30.9	1.52 (1.17–1.97)		21.6	29.5	1.38 (1.00–1.91)	19.5	32.0	1.64 (1.10–2.43)
41–50	16.7	17.5	Reference		17.2	18.4	Reference	16.2	16.8	Reference
51–60	15.3	11.7	0.77 (0.56–1.07)		15.7	12.4	0.70 (0.46–1.06)	15.0	11.1	0.87 (0.54–1.41)
61–70	17.5	6.08	0.26 (0.16–0.44)		17.3	5.3	0.23 (0.11–0.51)	17.6	6.7	0.28 (0.15–0.52)
≥71	16.3	6.45	0.26 (0.15–0.44)		16.4	6.7	0.25 (0.11–0.54)	16.2	6.3	0.26 (0.13–0.52)
Female	51.8	56.7	1.03 (0.82–1.29)	0.048	-	-	-	-	-	-
Education level	*n* = 21,722	*n* = 1758		<0.001	*n* = 10,437	*n* = 800		*n* = 11,285	*n* = 958	
High school or below	53.7	53.4	1.22 (0.99–1.48)		55.8	56.9	1.26 (0.98–1.63)	52.0	50.8	1.16 (0.85–1.58)
Two-year college graduate or technical school	19.3	22.3	1.24 (1.01–1.53)		10.8	12.6	1.13 (0.82–1.54)	27.4	29.8	1.28 (0.97–1.70)
University and above	26.1	23.0	Reference		32.0	29.4	Reference	20.6	18.2	Reference
Unknown	0.9	1.2	1.15 (0.50–2.66)		1.4	1.2	1.01 (0.36–3.12)	0.3	1.3	1.02 (0.35–3.51)
Employment status				<0.001						
Full-time employment/self-employed worker	42.2	45.3	Reference		60.4	61.8	Reference	25.3	32.8	Reference
Part-time employment	21.3	27.4	1.09 (0.83–1.41)		14.1	19.9	1.47 (1.02–2.12)	28.0	33.2	0.81 (0.57–1.16)
Retired/homemaker/student	24.1	18.4	1.05 (0.74–1.47)		9.1	6.1	1.44 (0.72–2.88)	38.1	27.8	0.80 (0.53–1.20)
Unemployed	12.4	8.8	1.03 (0.64–1.65)		16.5	12.2	1.30 (0.67–2.55)	8.5	6.3	0.77 (0.44–1.34)
Number of people in the household				<0.001						
1 (participant only)	14.7	22.2	Reference		14.5	27.1	Reference	15.0	18.5	Reference
2	33.3	24.4	0.84 (0.63–1.11)		32.9	21.0	0.62 (0.42–0.93)	33.6	26.9	1.12 (0.76–1.67)
3	25.4	22.5	0.78 (0.59–1.03)		25.0	24.1	0.76 (0.52–1.11)	25.7	21.2	0.87 (0.58–1.30)
≥4	26.6	31.0	0.97 (0.73–1.30)		27.6	27.9	0.74 (0.51–1.06)	25.8	33.4	1.39 (0.89–2.17)
Marital status				<0.0001						
Married	66.8	50.8	Reference		70.0	51.4	Reference	63.8	50.3	Reference
Unmarried	22.2	37.1	1.10 (0.84–1.43)		24.3	43.3	1.08 (0.77–1.52)	20.3	32.4	0.98 (0.66–1.46)
Divorced or bereaved	11.0	12.1	1.46 (1.00–2.17)		5.7	5.3	1.49 (0.93–2.37)	16.0	17.3	1.38 (0.81–2.35)
Annual household income † (Japanese yen)	*n* = 17,941	*n* = 1520		<0.0001	*n* = 9098	*n* = 724		*n* = 18,818	*n* = 643	
<1,000,000	2.4	6.7	3.60 (2.33–5.54)		1.9	6.3	3.66 (1.86–7.19)	2.8	7.0	4.51 (2.56–7.94)
≥1,000,000 & <5,000,000	36.5	47.1	2.11 (1.69–2.64)		35.9	46.2	1.99 (1.46–2.70)	37.1	47.9	2.43 (1.76–3.35)
≥5,000,000 & <10,000,000	32.1	24.4	Reference		36.6	28.8	Reference	28.0	47.9	Reference
≥10,000,000	8.6	5.4	0.91 (0.59–1.75)		10.7	8.4	1.02 (0.59–1.75)	6.6	3.1	0.67 (0.39–1.16)
Unknown	20.3	16.4	1.11 (0.84–1.46)		14.8	10.4	1.05 (0.73–1.62)	25.5	20.9	1.11 (0.88–1.62)
Public assistance status										
Number of types of public assistance				<0.001						
None	79.9	74.0	Reference		80.9	74.3	Reference	78.9	73.8	Reference
≥1	20.1	26.0	1.17 (1.00–1.47)		19.1	25.7	1.25 (1.03–1.71)	21.1	26.2	1.11 (1.03–1.54)
Types of public assistance										
Employment adjustment subsidy	3.8	7.0	1.63 (1.18–2.25)	<0.001	8.3	3.8	1.91 (1.21–3.02)	3.8	6.1	1.45 (0.93–2.26)
Public benefit for families with children	12.9	16.3	1.04 (0.80–1.37)	<0.001	10.7	12.5	0.99 (0.64–1.52)	14.4	22.9	1.45 (0.80–1.37)
Subsidy for sustaining business	2.4	3.9	1.98 (1.10–3.57)	0.002	3.0	4.3	1.72 (0.96–3.08)	1.8	3.6	1.98 (1.10–3.57)
Housing security Benefit	0.2	1.0	3.17 (1.18–8.53)	<0.001	0.30	1.6	3.55 (1.11–11.31)	0.1	0.6	2.47 (0.38–16.11)
Public assistance (welfare)	0.4	2.0	5.40 (2.24–12.97)	<0.001	0.5	2.8	3.90 (1.50–10.15)	0.3	1.3	7.83 (1.75–35.0)
Unemployment benefit	1.1	1.7	1.30 (0.77–2.22)	<0.001	1.0	2.7	2.58 (1.42–4.69)	1.2	0.9	0.62 (0.29–1.35)
Disability benefit	1.3	1.8	1.12 (0.67–1.90)	<0.001	1.6	2.1	0.96 (0.67–1.90)	1.0	1.6	1.25 (0.55–2.80)
Care allowance	0.2	0.2	1.21 (0.34–4.31)	0.109	0.2	0.1	0.46 (0.07–2.98)	0.2	0.1	2.08 (0.55–7.93)
Child rearing allowance	2.2	6.3	1.85 (1.15–2.97)	<0.001	1.6	3.8	2.34 (1.22–4.51)	2.8	8.3	1.61 (0.86–2.99)
Other allowance	5.3	8.4	1.92 (1.29–2.85)	<0.001	5.2	8.0	1.51 (0.88–2.60)	5.5	8.7	2.50 (1.43–4.37)
Severity of psychological distress (K6 score)				<0.001						
Normal (0–4)	69.8	31.0	Reference		73.4	34.8	Reference	66.4	28.1	Reference
Possible mild mood or anxiety disorder (5–9)	17.4	24.0	2.80 (2.19–3.59)		14.9	20.8	2.73 (1.90–3.93)	19.6	26.3	2.86 (2.04–4.01)
Mood or anxiety disorder (>10)	12.9	45.1	5.89 (4.74–7.33)		11.7	44.4	6.19 (4.61–8.31)	14.0	45.5	5.78 (4.18–7.98)

AOR: adjusted odds ratio, CI: confidence interval, FIES: Food Insecurity Experience Scale, K6: Kessler Psychological Distress Scale Values were weighted as percentages of variables. § ”Food security” was compared with “Food insecurity” in the weighted model for AOR. Sex, age, educational level, employment status, marital status, number of people in the household, annual household income, and number of types of public assistance status were mutually adjusted. * *p* values by the χ^2^ test. *p* < 0.05 was considered statistically significant. † Participants who answered “not willing to answer” or “unknown” (educational level: *n* = 96, annual household income: *n* = 4115) were excluded from the calculation. 1,000,000 yen was approximately equivalent to USD 6,929, 5,000,000 yen was approximately equivalent to USD 34,645, 10,000,000 yen was approximately equivalent to USD 69,2920, based on the exchange rate of 144.32 yen per USD in September 2022 [37].

## Data Availability

The original data presented in the study are openly available in JACSIS website (https://jacsis-study.jp/, accessed on 1 November 2025).

## Supplementary Materials

The following supporting information can be downloaded at https://www.mdpi.com/article/10.3390/nu17223536/s1, Table S1: Association between sociodemographic, socioeconomic, public assistance, and severity of psychological distress characteristics and Food Insecurity Experience Scale Score recommended by the FAO.

## Abbreviations

The following abbreviations are used in this manuscript:

FI	Food insecurity
FIES	Food Insecurity Experience Scale
FImod + sev	moderate-to-severe food insecurity
FIsev	severe food insecurity
AOR	adjusted odds ratio
CI	confidence interval
K6	Kessler Psychological Distress Scale
