# Supplementary material for: Associations of the Food Insecurity Experience Scale with Socioeconomic and Psychological Factors in Japan
Source: Nutrients. 2025 Nov 12;17(22):3536. doi: 10.3390/nu17223536 (PMC12655415; doi:10.3390/nu17223536)
Supplement: Supplementary file 1 [file nutrients-17-03536-s001.zip › nutrients-3952986-supplementary.pdf]

Table S1 Association between sociodemographic, socioeconomic, public assistance, severity of psychological distress characteristics and Food Insecurity Experience Scale Score recommended by the FAO * (n=23,576)

Values (%)	ALL	Food security /mild (0-3)	Food Moderate (4-6)	Food Severe (7-8)	AOR (95% CI)	*P*-value
N	23,576	22,829	389	358	-	
Number	-	96.83	1.65	1.52	-	
Number (weighted)	-	96.78	1.71	1.51	-	
Age						<0.0001
20-30	15.1	14.3	29.3	29.7	1.47 (0.88-2.48)	
31-40	21.4	21.1	27.9	26.7	0.98 (0.59-1.63)	
41-50	16.6	16.7	16.4	22.2	Reference	
51-60	14.6	15.2	13.2	10.1	0.46 (0.25-0.84)	
61-70	16.7	17.0	3.6	3.9	0.14 (0.52-1.38)	
≥71	15.4	15.8	9.5	7.4	1.90 (0.67-0.54)	
Female	52.2	52.2	57.2	46.6	0.69 (0.46-1.04)	0.017
Education level						0.064
High school or below	51.1	53.6	56.9	56.6	1.34 (0.91-1.98)	
Two-year college graduate or technical school	21.3	19.6	18.1	21.3	1.14 (0.69-1.90)	
University and above	27.2	26.0	24.6	20.7	Reference	
Unknown	0.4	0.9	0.4	1.4	1.91 (0.49-7.48)	
Employment status						<0.0001
Full-time employment/self-employed worker	44.4	42.4	38.8	49.0	Reference	
Part-time employment	20.8	21.6	27.9	28.0	0.89 (0.56-1.41)	
Retired/homemaker/student	23.8	23.9	21.0	12.3	0.79 (0.40-1.56)	
Unemployed	11.1	12.1	12.4	10.7	1.16 (0.61-2.23)	
Number of persons in household						<0.0001
1 (participant only)	19.1	15.0	23.6	29.1	Reference	
2	32.7	32.7	34.6	21.6	0.65 (0.40-1.06)	
3	24.6	25.3	18.4	21.7	0.65 (0.36-1.17)	
≥ 4	23.6	25.3	23.4	27.6	0.72 (0.43-1.21)	
Marital status						<0.0001
Married	64.8	66.2	43.5	46.6	Reference	
Unmarried	26.1	22.8	39.1	42.2	1.10 (0.73-1.66)	
Divorced/bereaved	9.2	11.0	17.4	11.1	1.67 (0.85-3.28)	
Annual household income (Japanese yen)						<0.0001
<1,000,000 (USD 6,931)	2.7	2.6	7.2	8.2	5.41 (2.89-10.15)	
≥ 1,000,000 & <5,000,000	36.0	36.7	59.1	52.3	2.93 (1.92-4.45)	
≥ 5,000,000 & <10,000,000	33.2	32.1	13.7	19.1	Reference	
≥ 10,000,000	10.6	8.5	7.3	3.5	0.77 (0.29-2.04)	
Unknown	17.6	20.2	12.7	17.0	1.74 (0.99-3.05)	
Public assistance status						<0.0001
Number of types of public assistance						
None	80.3	79.6	76.1	73.9	Reference	
≥ 1	19.7	20.5	23.8	26.2	1.17 (0.75-1.81)	
Types of public assistance						
Employment adjustment subsidy	3.9	3.9	7.1	8.2	1.84 (0.90-3.76)	<0.0001
Public benefit for families with children	12.4	12.9	18.8	13.3	1.06 (0.50-2.28)	0.005
Subsidy for sustaining business	2.3	2.5	2.8	4.0	1.20 (0.49-2.94)	<0.0001
Housing security benefit	0.2	0.2	0.1	1.6	4.91 (1.44-16.79)	<0.0001
Public assistance (welfare)	0.4	0.4	5.3	2.0	3.93 (1.26-12.22)	<0.0001
Unemployment benefit	1.2	1.1	2.2	1.3	1.00 (0.43-2.35)	<0.0001
Disability benefit	1.2	1.3	1.1	2.4	1.01 (0.47-2.18)	<0.0001
Care allowance	0.2	0.2	0.1	0.1	0.34 (0.04-2.60)	0.277
Child rearing allowance	2.1	2.4	8.2	6.1	2.75 (1.10-6.85)	0.001
Other allowance	5.0	5.4	13.8	6.1	0.64 (0.30-0.06)	<0.0001
Severity of psychological distress (K6 score)						<0.0001
Normal (0-4)	68.3	68.2	24.2	21.2	Reference	
possible mild mood or anxiety disorder (5-9)	16.7	17.8	18.3	17.5	3.80 (2.04-7.09)	
mood or anxiety disorder (>10)	15.0	14.0	57.6	61.4	11.81 (7.32-19.05)	

*'Food security /mild' was compared to 'Food Severe' in the weighted model for AOR. All the analysis were same as Table 4.
